# Is pre-operative haemoglobin A1c level a successful predictor of adverse outcome after cardiac surgery?

**DOI:** 10.1186/1749-8090-10-S1-A328

**Published:** 2015-12-16

**Authors:** Ai Hooe Tee, R Hasan, KE Mclaughlin, DJM Keenan, S Datta

**Affiliations:** 1 School of Medicine, University of Manchester, Oxford Rd, Manchester, M13 9PL, UK; 2 Department of Cardiac Surgery, Manchester Royal Infirmary, Oxford Road, Manchester, M139WL, UK

## Background/Introduction

Uncontrolled diabetes mellitus increases morbidity and mortality after cardiac surgery. Haemoglobin A1c (Hb A1c) is used to measure long-term glucose control. There have been reports of a higher incidence of wound infections with uncontrolled Hb A1c levels in patients undergoing cardiac surgery.

## Aims/Objectives

To establish clinical significance of elevated HbA1c level among patients who underwent cardiac surgery, and whether this may influence their postoperative mortality and morbidity.

## Method

We performed a retrospective review of patients who underwent cardiac surgery over a 4-year period (2012-2015). Patients were stratified into three HbA1c groups (I = HbA1c 20-41 mmol/mol; II = HbA1c 42-48 mmol/mol; III = HbA1c 49-150 mmol/mol). Study end points were post-operative wound infection, stroke and renal failure. Chi-square test and independent sample t-test were performed to compare variables of interest.

## Results

Among 1452 patients, 883 patients were in group I, 281 in group II and 288 in group III. There was statistically significant difference in post-operative wound infections between three groups (p < 0.006), with Group III having the highest rate of 7.6% while Group II 6.8% and Group I 3.5%. Patients with pre-operative HbA1c>42 mmol/mol (7.2% vs 3.5%) had a higher incidence of post-operative wound infections (p < 0.002; OR 2.134; 95% CI 1.322 - 3.445) when compared with Hb1Ac <42 mmol/mol. Patients with pre-operative HbA1c>;42 mmol/mol also had a significant increase in post-operative renal complications (p-value < 0.033; OR 2.569; 95%CI 1.049 - 6.290). Sub-group analysis among the urgent cases (n = 333) showed a 2.1 fold rise in wound infection (p < 0.02, OR 21, 95% CI 1.3-2.7). There was no statistical rise in incidence of stroke or mortality between the groups.

## Discussion/Conclusion

Elevated Hb1Ac was associated with increased wound infections and risk of renal dysfunction after cardiac surgery. Patients undergoing urgent surgery with undiagnosed diabetes may remain at increased risk of post-operative wound infections.

