# Erratum to: Is pre-operative haemoglobin A1c level a successful predictor of adverse outcome after cardiac surgery?

**DOI:** 10.1186/s13019-017-0586-4

**Published:** 2017-03-31

**Authors:** Ai Hooi Tee, R. Hasan, K. E. Mclaughlin, D. J. M. Keenan, S. Datta

**Affiliations:** 1 School of Medicine, University of Manchester, Oxford Rd, Manchester, M13 9PL UK; 2 Department of Cardiac Surgery, Manchester Royal Infirmary, Oxford Road, Manchester, M139WL UK

## Erratum

After the publication of this Meeting Abstract [1] it was noticed that the author name “Ai Hooi Tee” was misspelled as “Ai Hooe Tee”.
